# Impact of Caffeine Intake on Female Basketball Players’ Performance

**DOI:** 10.3390/nu17020235

**Published:** 2025-01-10

**Authors:** Raúl Nieto-Acevedo, Carlos García-Sánchez, Alfredo Bravo-Sánchez, Javier Abián-Vicén, Pablo Abián, Javier Portillo, Carlos Martínez-Rubio, Jorge Lorenzo Calvo, Javier Diaz-Lara

**Affiliations:** 1 Faculty of Sports Sciences, Universidad Alfonso X el Sabio, 28691 Villanueva de la Cañada, Spain; 2 Deporte y Entrenamiento Research Group, Facultad de Ciencias de la Actividad Física y del Deporte, Universidad Politécnica de Madrid, 28040 Madrid, Spain; c.gsanchez@upm.es; 3 Faculty of Health Sciences, Universidad Francisco de Vitoria, Ctra. Pozuelo-Majadahonda km 1800, 28223 Pozuelo de Alarcon, Spain; alfredo.bravo@ufv.es; 4 Performance and Sport Rehabilitation Laboratory, University of Castilla La Mancha, 45071 Toledo, Spain; javier.abian@uclm.es (J.A.-V.); luis.portillo@uclm.es (J.P.); javier.diazlara@uclm.es (J.D.-L.); 5 Faculty of Humanities and Social Sciences, Comillas Pontifical University, 28015 Madrid, Spain; pablo.abian@uclm.es; 6 Department of Education, Faculty of Education Sciences, University of Almería, 04120 Almería, Spain; carloscmrubio@gmail.com

**Keywords:** ergogenic aids, team sport, external load, supplementation, nutrition

## Abstract

Background: This study aimed to analyze the effect of caffeine ingestion on basketball performance in semi-professional female players. Methods: A double-blind, placebo-controlled, randomized experimental design was conducted, in two different periods separated by a week. Twelve female basketball players ingested 3 mg of caffeine/kg of body mass or a placebo. After 60 min, participants completed two repetitions of the specified sequence: Abalakov jump, countermovement jump (CMJ), 5-0-5 (505) test, and 20 m sprint. Later, external loads and game statistics were recorded during a 30-min simulated basketball game. Results: Significant improvements were observed in CMJ height and 505 test with caffeine ingestion compared to the placebo. Significant moderate differences were evident between conditions in external load variables, specifically, an increased number of high-intensity changes of direction during simulated games with caffeine ingestion. Two-point shot efficiency significantly improved after pre-caffeine ingestion compared with placebo. Conclusions: Three mg of caffeine per kg of body mass may serve as an effective ergogenic aid to increase physical performance and some variables of performance index in highly trained basketball players.

## 1. Introduction

Caffeine (1,3, 7-trymethylxantine) is widely used by elite athletes before and during competition in multiple sports disciplines [1,2,3]. Several investigations suggest that caffeine intake has an ergogenic effect on several aspects of physical performance, such as endurance exercise, [4,5] anaerobic-based exercise, and strength/power activities [6,7]. Furthermore, recent reviews and meta-analysis have shown that caffeine supplementation can also enhance physical performance in complex athletic disciplines requiring decision-making and high-intensity efforts, such as team or combat sports during specific performance tests and physical performance [8,9] and the success rate in sport-specific actions during competition [10].

The physical demands of basketball are characterized by high-intensity actions performed over very short periods and require high levels of speed, strength, and agility [11].

These movements are combined with different planes, angles, and specific basketball skills (e.g., layups, crossovers) [12]. Further efforts requiring high neuromuscular outputs, such as sprinting and jumping, are key predictors of success in team sports [13]. Thus, high change of direction performance is considered a particularly critical physical demand in basketball players [14]

The results of these two recent systematic reviews about caffeine and basketball report only ten studies published between 2000 and 2021 analyzing pre-exercise caffeine intake and only 30% of studies have been conducted on women [15,16]. These studies show that caffeine ingestion can enhance athletic performance, resulting in improved vertical jump height and faster running times at 10 and 20 m without the ball. Basketball performance metrics, including body impacts, free throws, rebounds, and assists, also showed improvement during simulated games. To the authors’ knowledge, only Puente et al. [17] and Raya-Gonzalez et al. [18] investigated the effects of caffeine supplementation on player activity using microsensor technology in simulated games.

This study explores how caffeine intake affects overall basketball performance in highly trained female players through specific tests and a simulated game. We hypothesize that consuming 3 mg of caffeine per kilogram of body mass before exercise will enhance physical performance in basketball-related tests and improve external load variables (player load, high impacts, dynamic stress load, and high-intensity changes of inertia). We also expect this intake to positively influence key performance stats, including field goal accuracy, rebounds, and assists, ultimately leading to better performance during the game.

## 2. Materials and Methods

### 2.1. Participants

The required sample size was calculated using G-Power software (version 3.1.7) with an effect size of 0.80, based on previous studies that reported effect sizes for the impact of caffeine on performance [17,18]. Therefore, the sample was 12 semi-professional female basketball players (age: 21.6 ± 3.8 years; weight: 68.8 ± 16.9 kg; height: 174.6 ± 9.8 cm) who volunteered to participate in this investigation. Players had previous experience in basketball (>10 years) and they trained for 3 h per day, 4 days a week, in addition to weekly matches, during the season in which this study was conducted.

The sample can be classified in the third tier of competition (highly trained or national level) following the classification provided by McKay et al. [19]. The exclusion criteria were taking medications or having any musculoskeletal injury that may compromise the trials. Moreover, following the ingestion of caffeine, the regular intake of the players could be considered as light-caffeine consumers (<100 mg/day). All players were informed about the details of this study and signed the consent form before the study began. Furthermore, the investigation adhered to the ethical guidelines of the Declaration of Helsinki and received approval from the Ethics Committee of the Universidad Alfonso X el Sabio (9/298—25 October 2024).

### 2.2. Pre-Experimental Procedures

The investigators recommended to the participants no ingestion of food or drinks with caffeine 24–48 prior experimental trials. In addition, it was required to avoid strenuous exercise and maintain their regular diet the day prior to each test.

### 2.3. Experimental Design

This study employed a double-blind, placebo-controlled, randomized, and counterbalanced experimental design. The randomization process was conducted using a computer software program that generates random sequences (Research Randomizer, www.randomizer.org). Participants had to perform two trials under the same experimental conditions. The conditions were at the same time (from 20 to 22 a.m.) and the same ambient conditions (23.5 *±* 0.8 °C temperature; 30.8 *±* 1.0 relative humidity).

The ingestion of caffeine was 3 mg per kg of body mass (99% purity, BulkPowders, Colchester, UK; women: 207.2 *±* 44.3 mg) in an opaque and unidentifiable capsule or an equal opaque capsule with cellulose (placebo). A caffeine or placebo capsule was ingested 60 min prior to each test as most research recommends ensuring proper absorption [20]. An external investigator, not present during the tests, dispensed the capsules labeled with alphanumeric codes, ensuring that both participants and researchers were blinded to the capsules consumed by each group. The code assigned to each participant was revealed after the statistical analysis.

### 2.4. Experimental Protocol

Participants were required to arrive 75 min before the tests, then they took the corresponding capsule—caffeine or placebo. After the capsule ingestion, players did a standardized warm-up, doing 5 min of low aerobic and mobility exercises, followed by 10 min of basketball drill, and 3 min of high-intensity actions.

Following the warm-up, players rested for 3 min and underwent a series of fitness tests, which were conducted in the following sequence: 2 repetitions of the Abalakov jump (AJ) and 2 repetitions of the countermovement jump (CMJ) using ForceDecks FD4000 Dual Force Platforms (Vald Performance, Brisbane, Queensland, Australia). To minimize fatigue-induced performance changes, each jump was separated by a 10–15 s rest interval, followed by 2 repetitions of the 5-0-5 test and 2 repetitions of the 20-m sprint test. For the 5-0-5 test, a pair of photoelectric cells (Polifemo Light Radio; Microgate, Bolzano, Italy) were used and the players were instructed to accelerate as fast as possible before the deceleration. After touching the turning line, they were required to return to the starting gates. For the 20 m sprint test, players completed maximal 20-m sprints with performance time assessed using 6 pairs of photoelectric cells (Polifemo Light Radio; Microgate. Bolzano. Italy) at 0, 10, and 20 m. Players started each sprint 0.5 m before the first timing gate and commenced each sprint of their own volition. To encourage players to give their highest potential, investigators gave verbal feedback and instructions. Two minutes of rest was given between each 20 m sprint and 5-0-5 test.

After fitness testing, a simulated game (3 quarts of 10 min with 2 min of rest between each one) was played following the rules of the International Basketball Federation (FIBA; except for the game duration) and on an official basketball court. The matches were supervised by a couple of referees. The same teams were maintained on the two occasions. Previously, technical staff created two balance teams to avoid big differences in the games. Each team was composed of six players, then each 5 min a player of each team was changed to rest. For this reason, and due to the shorter duration of the simulated game, they did not have time-outs. Moreover, game systems and zone defense were not allowed. To record games, a video camera was used (JVC GZ-R315BEU Full HD video camera. JVC^®^, Tokyo, Japan). To ensure the blinding process of video data analysis, two investigators individually examined the matches. They conducted a notational analysis of the plays of each player in both matches. The following game-related statistics were collected: total points during the game, two- and three-point field goals (made, attempted, and accuracy), free throws, offensive, defensive, total number of rebounds, assists, steals, turn-overs, received and committed blocks, dunks, and received and committed fouls. These variables were used to calculate the performance index rating with the following formula: (points + total rebounds + assists + steals + blocks committed + fouls received) − (missed shots + turnovers + fouls committed) used by the FIB [21].

Additionally, all the players were monitored using an electronic position device (WIMU Pro, Hudl^®^ SL, Lincoln, NE, USA) with a sampling of 100 Hz. This device is equipped with four accelerometers that record data across different magnitude ranges (x2: 16 G; x1: 32 G; x1: 400 G at 1000 Hz), three gyroscopes (x2: ± 2000 degrees per second; x1: ±4000 degrees per second at 1000 Hz), a magnetometer (±8 Gauss at 160 Hz), and one barometer (±1200 mbar at 100 Hz). Moreover, each device has a number affiliated with the same player for both days to limit problems that may exist due to the inter-unit reliability of the devices. After the simulated matches, the files were exported to a USB memory and analyzed using the manufacturer’s specific software (SPRO. version 2.2.0. Hudl^®^ SL, Lincoln, USA). Finally, data was exported to an Excel spreadsheet and introduced into a statistical program for analysis. The external load metrics used during simulated games were: (1) PlayerLoad (PL) expressed in arbitrary units (a.u.) and corresponding with the square root of the sum of the squared acceleration on the three planes divided by 100 [22]; (2) Dynamic Stress Load (DSL), expressed in arbitrary units (a.u.) and defined as the sum of the weighted acceleration-based impacts over 2G [23]; (3) +2G-PL; expressed in arbitrary units (a.u.) and defined as the accumulation of PL above 2G; (4) +4G-PL, expressed in arbitrary units (a.u.) and defined as the accumulation of PL above 4G; (5) High-Intensity Changes of Inertia (HI COI), calculated as the sum of accelerations greater than 1.25G projected onto the horizontal plane [24] and represented based on their direction (e.g., Front-Left HI COI) or as the total accelerations (SUM HI COI); (6) SUM HI COI lateral, calculated as the sum of COI projected only in the mediolateral planes; and (7) High Impacts, calculated as the sum of impacts higher than 8G.

Once the test was finished, participants were required to complete a questionnaire about how they felt in terms of muscle condition, endurance, and perceived effort. Each item was evaluated on a 1–10-point scale used under the same conditions by other researchers [25]. Additionally, participants completed a survey the following morning to assess sleep quality, feelings of nervousness, gastrointestinal issues, and other discomforts related to caffeine consumption. This survey consisted of seven yes/no questions and has been previously used to evaluate side effects associated with caffeine capsules [26].

### 2.5. Statistical Analysis

Descriptive statistics are displayed as mean ± standard deviation. First, to check the normality of the variables, the Shapiro–Wilk test was used (*p* > 0.05). Secondly, Student’s *t*-test were used to analyze differences between experimental conditions for each dependent variable. The McNemar test was employed to identify changes in the frequency of side effects reported following each condition. Cohen’s d was used for effect size and followed the scale previously reported: trivial (<0.19), small (0.20–0.49), medium (0.50–0.79), and large (>0.80). The significance level was fixed at *p* < 0.05. The 95% confidence interval for the mean difference (95% CI) between both conditions was analyzed. Data analysis was conducted using JAMOVI (version 2.3.21.0. 2022) for Macintosh.

## 3. Results

**Figure 1:**
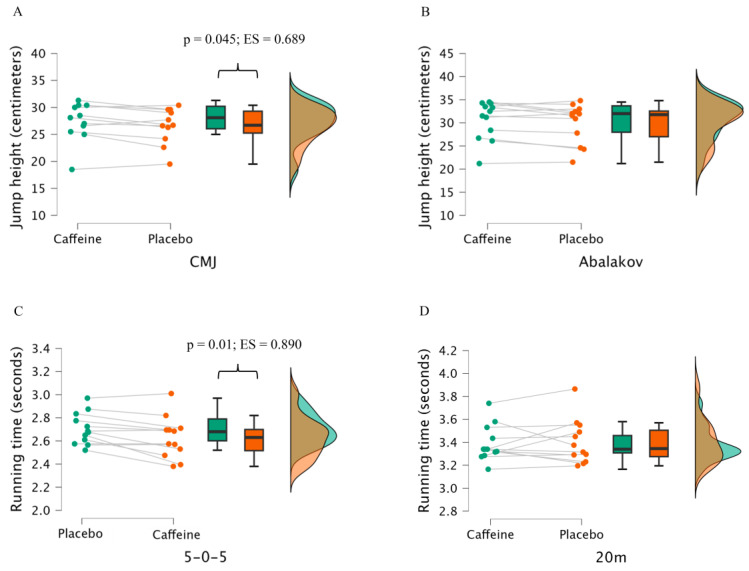
Raincloud plots of jump height during countermovement jump (CMJ) (**A**), Abalakov jump (**B**), and running time during 505 test (**C**) and 20 m test (**D**), with the ingestion of caffeine (3 mg of caffeine per kg of body mass) or a placebo. Data are the mean ± standard deviation for 12 female basketball players. The whisker box represents the distribution, and the middle line and bars represent the median, 95% CI, and SD of the given group. The raincloud plots represent the overlapped distributions of the 2 groups.

The results of the fitness test comparing caffeine and placebo can be found in Figure 1. Players under caffeine ingestion showed significant moderate-large performance in CMJ height (26.68 ± 3.21 cm vs. 27.88 ± 3.84 cm; *p* = 0.035, ES = 0.695, medium), and 505 test (2.63 ± 0.18 s vs. 2.71 ± 0.14 s; *p* = 0.01, ES = 0.890, large) compared to the placebo.

Table 1 shows the mean ± SD of caffeine and placebo groups in external loads variables during matches. Significant, moderate differences were clear between groups in Left HI COI, Front-Right HI COI, Front-Left HI COI, Back-Left HI COI, SUM HI COI, and SUM HI COI-Lateral variables during simulated games (*p* < 0.05, ES range = 0.45–0.55, medium).

Table 2 presents game-related statistics examined during the simulated: total points during the game, two- and three-point field goals (made, attempted, and accuracy), free throws, offensive, defensive, and total number of rebounds, assists, steals, turn-overs, received and committed blocks, dunks, and received and committed fouls. The pre-exercise caffeine ingestion significantly improved the accuracy in 2-point field goals percentage (d = 0.76; *p* = 0.031). The performance index rating was also higher for the caffeine condition, but without significant differences between groups (d = 0.60; *p* = 0.062). In relation to the other game-related variables, no significant differences were found (*p* > 0.05).

The day after the test, players reported a significantly superior prevalence of activeness (7.0 vs. 33.0%; *p* < 0.05) and tachycardia (7.0 vs. 22.0%; *p* < 0.05), while the placebo group showed significantly higher urine output (20 vs. 0%; *p* < 0.05) than the caffeine group. However, the remaining side effects (nervousness, irritability, activeness, gastrointestinal discomforts, headache, and muscle pain) were similar between both conditions (*p* > 0.05; Table 3).

## 4. Discussion

Our aim in the present study was to analyze the effectiveness of caffeine (3 mg/kg) to improve on different fitness tests and in-game activity monitoring in female basketball players. Briefly, the main results showed acute caffeine supplementation promoted significant moderate-very large improvements in vertical jump, change-of-direction, and an increased number of high-intensity changes of directions during simulated games. These effects were accompanied by higher accuracy during two-point field goals. Our findings indicate that a caffeine dose of 3 mg/kg is an effective ergogenic aid to enhance physical and game performance in female basketball players.

Previous studies have reported trivial-small increases in jump height with caffeine ingestion in male, female, and young basketball players [17,18,27,28]. We also observed significantly moderate CMJ improvements with caffeine ingestion compared to the placebo. In addition, similar to Raya-González et al. [18], we also found a significant enhancement in change-of-direction speed (e.g., 505 test) in favor of caffeine ingestion compared to the placebo. Nevertheless, both studies [17,28] did not find significant differences in change-of-direction speed between caffeine and placebo in semi-professional male and professional female basketball players. Thus, given that these studies provided contrasting findings, further research is required to investigate the effect of caffeine intake on change of direction test in basketball players.

Two studies examined the effects of caffeine supplementation on in-game demands on semi-professional male, and professional female basketball players [17,18]. In the study by Puente et al. [17], trivial improvements were observed in the number of light impacts (≤0.99 G) for the caffeine group during simulated basketball games. In this line, Raya-González et al. [18] showed non-significant, trivial changes in most activity variables (distance, speed, and acceleration variables) during simulated games with acute caffeine (6 mg/kg) ingestion compared to the placebo trial. Notably, the present study incorporated new algorithm-based metrics through inertial measurement units (IMUs), which allowed the capture of specific basketball actions during games, such as changes of direction/inertia [24]. The results of Espasa-Labrador et al. [29] observed that IMUs offer important information about high-intensity movements and patterns, showing a strong link between physical workload and player performance in women’s basketball. While this previous research [18] indicates that caffeine consumption may not have a significant impact on high-intensity actions performed during basketball games, our findings suggest the possibility that the performance improvements observed in fitness tests (such as the 5-0-5 agility test) with caffeine intake could translate into enhanced performance in game situations. This raises new considerations not previously considered, particularly regarding caffeine’s impact on high-intensity direction changes during a basketball game.

In relation to basketball statistics, Puente et al. [17] found that the performance index rating proposed by FIBA, was significantly better when players consumed caffeine. However, our findings regarding the performance index rating revealed no significant differences between the placebo and caffeine conditions (*p* = 0.062). When we examine the game-related statistics individually, Puente et al. [17] observed that caffeine intake resulted in more free throws attempted and made by both male and female players during simulated games, but they did not find differences in accuracy for two-point and three-point field goals or free throws. In contrast, our investigation shows improvements in the two-point field goal percentage (*p* = 0.01) (see Table 2). This positive aspect of caffeine’s effect on game performance is consistent with existing research highlighting caffeine’s beneficial effects. For instance, pre-competition caffeine consumption in male and female volleyball players enhanced the proportion of successful actions related to serves, receptions, spikes, and digs without increasing player errors [30]. Additionally, Diaz-Lara et al. [10], in a recent meta-analysis, indicated that caffeine might improve the proportion of positive actions during matches and games in various team sports. In our study, one possible explanation for the improved effectiveness of two-point shots following caffeine ingestion could be linked to enhanced physical performance, which could facilitate better positioning for executing these sport-specific actions.

A side effects questionnaire was administered in the morning following testing to determine whether caffeine represents a risk to the health of basketball players. The post-test questionnaire about self-performance and the prevalence of side effects indicated that a higher prevalence of tachycardia and activeness was observed after caffeine supplementation compared to the placebo. The frequency of the rest of the side effects was very similar between both conditions, placebo or caffeine. The higher frequency of tachycardia and activeness after caffeine intake should be considered when recommending caffeine to increase performance in female team sports.

The findings of this study offer valuable insights for nutritionists, coaches, and athletic trainers working with semi-professional female basketball players. Nutritionists may consider recommending a caffeine dosage of 3 mg/kg of body mass as part of the athletes’ preparation, particularly during matches. This recommendation is based on caffeine’s positive effects on agility, high-intensity efforts, and shot accuracy, which can be beneficial during critical moments in a game and may enhance overall performance. However, it is essential to evaluate the individual effects of caffeine before its use in competition, as responses can vary among athletes. We suggest personalized caffeine intake, considering individual tolerance, arousal, side effects, health status, and potential sensitivities.

## 5. Limitations and Directions for Further Research

While this study presents novel findings regarding the impact of acute caffeine supplementation on performance and in-game activity in basketball players, it is crucial to consider certain limitations that could affect the generalizability and interpretation of the results. Firstly, the sample size is relatively small, with only 12 participants, all of whom were semi-professional players. The small sample size limits the generalizability of the results, as the findings may not apply to a broader population, such as recreational athletes or those at different competition levels. The sample in this study was limited to female players, which limits our ability to compare the effects of caffeine between genders. However, we encourage further research on women, as only 34% [10] of such studies focus on this gender, which represents half of the world’s population.

Another limitation is that the teams were intentionally created to have comparable athletic and technical skills. In addition, each player was substituted after 5 min to ensure that the time played by each participant was identical, whereas the substitutions in official basketball games are unlimited. Consequently, the playing durations and physical demands in this study were likely greater than those experienced in an official basketball game.

## 6. Conclusions

In summary, consuming 3 mg/kg of caffeine before exercise improved performance in specific tests, including jump height during the countermovement jump and the 505-agility test. It also improves the accuracy of two throws and high-intensity directional changes in simulated games. These findings suggest a practical ergogenic effect in enhancing physical performance in basketball. Given the benefits and potential side effects, we encourage basketball coaches, trainers, and nutritionists to test caffeine use in simulated and real match scenarios. Combining technical–tactical analyses with physical performance assessments allows them to personalize caffeine intake recommendations for each player.

## Figures and Tables

**Table 1:** Mean ± standard deviation and differences in external workloads achieved between placebo and caffeine conditions in female basketball players during simulated games.

External Load Variable	Placebo			Caffeine			Diff.		95%CI		Cohen’s d	*p* Value
High Impacts (n)	13.5	±	17.3	16.1	±	21.4	−2.6	−10.5	to	5.3	−0.13	0.52
Total Jumps (n)	8.5	±	7.4	9.3	±	7.9	−0.8	−4.0	to	2.2	−0.11	0.59
Jumps >3G (n)	3.1	±	3.00	3.8	±	3.3	−0.7	−2.0	to	0.6	−0.20	0.32
Player Load (a.u.)	19.1	±	12.3	19.5	±	12.7	−0.4	−5.5	to	4.6	−0.03	0.87
DSL (a.u.)	40.3	±	47.7	43.8	±	50.9	−3.5	−23.5	to	16.5	−0.07	0.73
>2G PL (a.u.)	6.2	±	4.5	6.4	±	4.7	−0.2	−2.1	to	1.6	−0.05	0.82
>4G PL (a.u.)	1.6	±	1.5	1.7	±	1.6	−0.1	−0.7	to	0.5	−0.07	0.74
F high COI (n)	0.3	±	0.5	0.3	±	0.6	−0.0	−0.3	to	0.2	−0.08	0.70
B high COI (n)	0.1	±	0.3	0.2	±	0.7	−0.1	−0.3	to	0.1	−0.19	0.36
R high COI (n)	0.5	±	1.1	1.0	±	1.6	−0.4	−1.0	to	0.1	−0.31	0.14
L high COI (n)	0.3	±	0.6	1.0	±	2.34	−0.8	−14.4	to	−0.1	−0.44	0.04 *
F-R high COI (n)	0.1	±	0.3	0.5	±	1.6	−0.5	−0.8	to	−0.1	−0.55	0.01 *
F-L high COI (n)	0.0	±	0.2	0.4	±	1.2	−0.4	−0.7	to	−0.0	−0.45	0.03 *
B-R high COI (n)	0.0	±	0.2	0.2	±	0.6	−0.2	−0.4	to	0.0	−0.36	0.08 *
B-L high COI (n)	0.0	±	0.0	0.3	±	0.8	−0.3	−0.5	to	−0.1	−0.54	0.01 *
SUM HI COI (n)	1.3	±	1.8	3.9	±	7.2	−2.6	−4.7	to	−0.5	−0.5	0.02 *
SUM HI COI lateral (n)	1.0	±	1.5	3.5	±	6.4	−2.5	−4.3	to	−0.6	−0.53	0.01 *

(*) Different from placebo (*p* < 0.05). Diff. = mean difference between caffeine and placebo. 95% CI = 95% confidence interval for the mean difference. G = gravitational; DSL = Dynamic Stress Load; F = Front; B = Back; R = Right; L = Left; COI = Changes of Inertia; HI = High Intensity; SUM = summation; a.u = arbitrary units.

**Table 2:** Game-related statistics with the ingestion of caffeine (3 mg of caffeine per kg of body mass) or a placebo. Data are mean ± standard deviation for 12 female basketball players.

Game Statistic	Placebo			Caffeine			Diff.	95% CI			*p* Value	Cohen’s d
Points	4.4	±	3.6	6.6	±	4.8	−2.2	−4.9	to	0.6	0.11	−0.50
2-point field goals made	1.8	±	1.8	2.3	±	1.7	−0.6	−2.0	to	0.8	0.37	−0.27
2-point field goals attempted	5.5	±	3.5	4.9	±	3.6	0.6	−2.4	to	3.5	0.67	0.13
Accuracy in 2-point field goals (%)	26.9	±	21.1	49.7	±	22.6	−22.8	−38.2	to	−7.4	0.01 *	−0.94
3-point field goals made	0.2	±	0.4	0.5	±	1.0	−0.3	−1.1	to	0.4	0.34	−0.29
3-point field goals attempted	2.1	±	1.9	2.0	±	1.8	0.1	−1.8	to	2.0	0.93	0.03
Accuracy in 3-point field goals (%)	4.2	±	10.4	12.5	±	22.6	−8.3	−25.5	to	8.9	0.31	−0.31
Free throws made	0.4	±	0.7	0.4	±	0.8	0.0	−0.5	to	0.5	1.00	0.00
Free throws attempted	1.2	±	1.9	1.1	±	1.3	−8.3	−1.4	to	1.6	0.91	0.04
Accuracy in free throws (%)	12.8	±	20.3	16.7	±	32.6	−3.9	−24.3	to	16.5	0.68	−0.12
Offensive rebounds	3.3	±	2.3	3.8	±	2.8	−0.4	−3.2	to	2.4	0.75	−0.10
Defensive rebounds	1.2	±	1.3	0.8	±	1.2	0.4	−0.9	to	1.7	0.49	0.21
Total rebounds	4.3	±	3.4	4.4	±	3.1	−0.2	−3.5	to	3.1	0.91	−0.03
Assists	1.0	±	1.3	0.8	±	1.2	0.3	−0.8	to	1.3	0.60	0.16
Steals	1.0	±	0.9	1.3	±	1.2	−0.3	−1.4	to	0.7	0.50	−0.20
Turnovers	2.1	±	1.2	1.5	±	1.8	0.6	−1.0	to	2.2	0.44	0.23
Blocks committed	0.3	±	0.7	0.1	±	0.3	0.3	−0.2	to	0.7	0.28	0.33
Blocks received	0.2	±	0.4	0.3	±	0.5	−8.3	−0.5	to	0.3	0.674	−0.13
Fouls committed	0.7	±	0.8	0.7	±	0.7	0.0	−0.6	to	0.6	1.00	0.00
Fouls received	0.9	±	1.1	0.7	±	0.9	0.3	−0.6	to	1.1	0.51	0.19
Performance index rating	2.6	±	6.8	6.7	±	5.7	−4.1	−8.4	to	0.2	0.06	−0.60

(*) Different from placebo (*p* < 0.05). Diff. = mean difference between caffeine and placebo. 95% CI = 95% confidence interval for the mean difference.

**Table 3:** Prevalence of side effects (%) in placebo and caffeine conditions in female basketball players.

Side Effect	Placebo	Caffeine
Insomnia	13	11
Tachycardia	7	22 *
Anxiety	0	0
Abdominal discomfort	0	0
Headache	0	11
Activeness	7	33 *
Urine output	20	0 *

(*) Different from placebo (*p* < 0.05).

## Data Availability

The original contributions presented in this study are included in the article. Further inquiries can be directed to the corresponding author.

## References

[1] Jiménez S.L., Díaz-Lara J., Pareja-Galeano H., Del Coso J., Jimenez S.L., Diaz-Lara J., Pareja-Galeano H., Del Coso J. (2021). Caffeinated Drinks and Physical Performance in Sport: A Systematic Review. Nutrients.

[2] Grgic J., Grgic I., Pickering C., Schoenfeld B.J., Bishop D.J., Pedisic Z. (2019). Wake up and Smell the Coffee: Caffeine Supplementation and Exercise Performance—An Umbrella Review of 21 Published Meta-Analyses. Br. J. Sports Med..

[3] Aguilar-Navarro M., Muñoz G., Salinero J.J., Muñoz-Guerra J., Fernández-álvarez M., Plata M.D.M., Del Coso J. (2019). Urine Caffeine Concentration in Doping Control Samples from 2004 to 2015. Nutrients.

[4] Southward K., Rutherfurd-Markwick K.J., Ali A. (2018). The Effect of Acute Caffeine Ingestion on Endurance Performance: A Systematic Review and Meta-Analysis. Sports Med..

[5] Souza D.B., Del Coso J., Casonatto J., Polito M.D. (2017). Acute Effects of Caffeine-Containing Energy Drinks on Physical Performance: A Systematic Review and Meta-Analysis. Eur. J. Nutr..

[6] Grgic J., Trexler E.T., Lazinica B., Pedisic Z. (2018). Effects of Caffeine Intake on Muscle Strength and Power: A Systematic Review and Meta-Analysis. J. Int. Soc. Sports Nutr..

[7] Grgic J., Varovic D. (2022). Ergogenic Effects of Caffeine on Ballistic (Throwing) Performance: A Meta-Analytical Review. Nutrients.

[8] Diaz-Lara J., Grgic J., Detanico D., Botella J., Jimenez S.L., Del Coso J. (2022). Effects of Acute Caffeine Intake on Combat Sports Performance: A Systematic Review and Meta-Analysis. Crit. Rev. Food Sci. Nutr..

[9] Salinero J.J., Lara B., Del Coso J. (2019). Effects of Acute Ingestion of Caffeine on Team Sports Performance: A Systematic Review and Meta-Analysis. Res. Sports Med..

[10] Diaz-Lara J., Nieto-Acevedo R., Abian-Vicen J., Coso J. (2024). Del Can Caffeine Change the Game? Effects of Acute Caffeine Intake on Specific Performance in Intermittent Sports During Competition: A Systematic Review and Meta-Analysis. Int. J. Sports Physiol. Perform..

[11] Stojanović E., Stojiljković N., Scanlan A.T., Dalbo V.J., Berkelmans D.M., Milanović Z. (2018). The Activity Demands and Physiological Responses Encountered During Basketball Match-Play: A Systematic Review. Sports Med..

[12] Taylor J.B., Wright A.A., Dischiavi S.L., Townsend M.A., Marmon A.R. (2017). Activity Demands During Multi-Directional Team Sports: A Systematic Review. Sports Med..

[13] Harper D.J., Carling C., Kiely J. (2019). High-Intensity Acceleration and Deceleration Demands in Elite Team Sports Competitive Match Play: A Systematic Review and Meta-Analysis of Observational Studies. Sports Med..

[14] Sugiyama T., Maeo S., Kurihara T., Kanehisa H., Isaka T. (2021). Change of Direction Speed Tests in Basketball Players: A Brief Review of Test Varieties and Recent Trends. Front. Sports Act. Living.

[15] Lazić A., Kocić M., Trajković N., Popa C., Peyré-Tartaruga L.A., Padulo J. (2022). Acute Effects of Caffeine on Overall Performance in Basketball Players-A Systematic Review. Nutrients.

[16] Tan Z.S., Sim A., Kawabata M., Burns S.F. (2022). A Systematic Review of the Effects of Caffeine on Basketball Performance Outcomes. Biology.

[17] Puente C., Abian-Vicen J., Salinero J.J., Lara B., Areces F., Del Coso J. (2017). Caffeine Improves Basketball Performance in Experienced Basketball Players. Nutrients.

[18] Raya-Gonzalez J., Scanlan A.T., Soto-Celix M., Rodriguez-Fernandez A., Castillo D. (2021). Caffeine Ingestion Improves Performance During Fitness Tests but Does Not Alter Activity During Simulated Games in Professional Basketball Players. Int. J. Sports Physiol. Perform..

[19] McKay A.K.A., Stellingwerff T., Smith E.S., Martin D.T., Mujika I., Goosey-Tolfrey V.L., Sheppard J., Burke L.M. (2021). Defining Training and Performance Caliber: A Participant Classification Framework. Int. J. Sports Physiol. Perform..

[20] Graham T.E., Spriet L.L. (1995). Metabolic, Catecholamine, and Exercise Performance Responses to Various Doses of Caffeine. J. Appl. Physiol. (1985).

[21] Arrieta H., Torres-Unda J., Gil S.M., Irazusta J. (2016). Relative Age Effect and Performance in the U16, U18 and U20 European Basketball Championships. J. Sports Sci..

[22] Gómez-Carmona C.D., Bastida-Castillo A., González-Custodio A., Olcina G., Pino-Ortega J. (2020). Using an Inertial Device (WIMU PRO) to Quantify Neuromuscular Load in Running: Reliability, Convergent Validity, and Influence of Type of Surface and Device Location. J. Strength. Cond. Res..

[23] Beato M., De Keijzer K.L., Carty B., Connor M. (2019). Monitoring Fatigue during Intermittent Exercise with Accelerometer-Derived Metrics. Front. Physiol..

[24] Avilés R., Souza D.B., Pino-Ortega J., Castellano J. (2023). Assessment of a New Change of Direction Detection Algorithm Based on Inertial Data. Sensors.

[25] Salinero J.J., Lara B., Abian-Vicen J., Gonzalez-Millán C., Areces F., Gallo-Salazar C., Ruiz-Vicente D., Del Coso J. (2014). The Use of Energy Drinks in Sport: Perceived Ergogenicity and Side Effects in Male and Female Athletes. Br. J. Nutr..

[26] Diaz-Lara F.J., Del Coso J., García J.M., Portillo L.J., Areces F., Abián-Vicén J. (2016). Caffeine Improves Muscular Performance in Elite Brazilian Jiu-Jitsu Athletes. Eur. J. Sport. Sci..

[27] Abian-Vicen J., Puente C., Salinero J.J., González-Millán C., Areces F., Muñoz G., Muñoz-Guerra J., Del Coso J. (2014). A Caffeinated Energy Drink Improves Jump Performance in Adolescent Basketball Players. Amino Acids.

[28] Stojanovic E., Scanlan A.T., Milanovic Z., Fox J.L., Stankovic R., Dalbo V.J., Stojanović E., Scanlan A.T., Milanović Z., Fox J.L. (2022). Acute Caffeine Supplementation Improves Jumping, Sprinting, and Change-of-Direction Performance in Basketball Players When Ingested in the Morning but Not Evening. Eur. J. Sport. Sci..

[29] Espasa-Labrador J., Martínez-Rubio C., Oliva-Lozano J.M., Calleja-González J., Carrasco-Marginet M., Fort-Vanmeerhaeghe A. (2024). Relationship between Physical Demands and Player Performance in Professional Female Basketball Players Using Inertial Movement Units. Sensors.

[30] Del Coso J., Pérez-López A., Abian-Vicen J., Salinero J.J., Lara B., Valadés D. (2014). Enhancing Physical Performance in Male Volleyball Players with a Caffeine-Containing Energy Drink. Int. J. Sports Physiol. Perform..

